# Supplementary material for: OpenMM 7: Rapid development of high performance algorithms for molecular dynamics
Source: PLoS Comput Biol. 2017 Jul 26;13(7):e1005659. doi: 10.1371/journal.pcbi.1005659 (PMC5549999; doi:10.1371/journal.pcbi.1005659)
Supplement: S1 Source Code — (ZIP) [file pcbi.1005659.s002.zip › OpenMM-7.0.1-Source/docs/api-python/generated/simtk.openmm.openmm.State.html]

State — OpenMM 7.0.1.dev-5e86c4f documentation


# State¶

*class* `simtk.openmm.openmm.``State`(*simTime=None*, *energy=None*, *coordList=None*, *velList=None*, *forceList=None*, *periodicBoxVectorsList=None*, *paramMap=None*)¶
:   A State object records a snapshot of the
    current state of a simulation at a point
    in time. You create it by calling
    getState() on a Context.

    When a State is created, you specify what
    information should be stored in it. This
    saves time and memory by only copying in
    the information that you actually want.
    This is especially important for forces
    and energies, since they may need to be
    calculated. If you query a State object
    for a piece of information which is not
    available (because it was not requested
    when the State was created), it will
    return None.

    In general return values are Python Units
    (https://simtk.org/home/python\_units).
    Among other things Python Units provides a
    container class, Quantity, which holds a
    value and a representation of the value’s
    unit. Values can be integers, floats,
    lists, numarrays, etc. Quantity objects
    can be used in arithmetic operation just
    like number, except they also keep track
    of units. To extract the value from a
    quantity, us the value\_in\_unit() method.
    For example, to extract the value from a
    length quantity, in units of nanometers,
    do the following:
    myLengthQuantity.value\_in\_unit(unit.nanometer)

    `__init__`(*simTime=None*, *energy=None*, *coordList=None*, *velList=None*, *forceList=None*, *periodicBoxVectorsList=None*, *paramMap=None*)¶

    Methods

    |  |  |
    | --- | --- |
    | `__init__`([simTime, energy, coordList, ...]) |  |
    | `getForces`([asNumpy]) | Get the force acting on each particle with units. |
    | `getKineticEnergy`() | Get the total kinetic energy of the system with units. |
    | `getParameters`() | Get a map containing the values of all parameters. |
    | `getPeriodicBoxVectors`([asNumpy]) | Get the three periodic box vectors if this state is from a simulation using PBC . |
    | `getPeriodicBoxVolume`() | Get the volume of the periodic box. |
    | `getPositions`([asNumpy]) | Get the position of each particle with units. |
    | `getPotentialEnergy`() | Get the total potential energy of the system with units. |
    | `getTime`() | Get the time for which this State was created. |
    | `getVelocities`([asNumpy]) | Get the velocity of each particle with units. |

    `getTime`()¶
    :   Get the time for which this State was created.

    `getPeriodicBoxVectors`(*asNumpy=False*)¶
    :   Get the three periodic box vectors if this state is from a
        simulation using PBC .

    `getPeriodicBoxVolume`()¶
    :   Get the volume of the periodic box.

    `getPositions`(*asNumpy=False*)¶
    :   Get the position of each particle with units.
        Raises an exception if postions where not requested in
        the context.getState() call.
        Returns a list of tuples, unless asNumpy is True, in
        which case a Numpy array of arrays will be returned.
        To remove the units, divide return value by unit.angstrom
        or unit.nanometer. See the following for details:
        https://simtk.org/home/python\_units

    `getVelocities`(*asNumpy=False*)¶
    :   Get the velocity of each particle with units.
        Raises an exception if velocities where not requested in
        the context.getState() call.
        Returns a list of tuples, unless asNumpy is True, in
        which case a Numpy array of arrays will be returned.
        To remove the units, you can divide the return value by
        unit.angstrom/unit.picosecond or unit.meter/unit.second,
        etc. See the following for details:
        https://simtk.org/home/python\_units

    `getForces`(*asNumpy=False*)¶
    :   Get the force acting on each particle with units.
        Raises an exception if forces where not requested in
        the context.getState() call.
        Returns a list of tuples, unless asNumpy is True, in
        which case a Numpy array of arrays will be returned.
        To remove the units, you can divide the return value by
        unit.kilojoule\_per\_mole/unit.angstrom or
        unit.calorie\_per\_mole/unit.nanometer, etc.
        See the following for details:
        https://simtk.org/home/python\_units

    `getKineticEnergy`()¶
    :   Get the total kinetic energy of the system with units.
        To remove the units, you can divide the return value by
        unit.kilojoule\_per\_mole or unit.calorie\_per\_mole, etc.
        See the following for details:
        https://simtk.org/home/python\_units

    `getPotentialEnergy`()¶
    :   Get the total potential energy of the system with units.
        To remove the units, you can divide the return value by
        unit.kilojoule\_per\_mole or unit.kilocalorie\_per\_mole, etc.
        See the following for details:
        https://simtk.org/home/python\_units

    `getParameters`()¶
    :   Get a map containing the values of all parameters.

    `__delattr__`¶
    :   x.\_\_delattr\_\_(‘name’) <==> del x.name

    `__format__`()¶
    :   default object formatter

    `__getattribute__`¶
    :   x.\_\_getattribute\_\_(‘name’) <==> x.name

    `__hash__`¶

    `__reduce__`()¶
    :   helper for pickle

    `__reduce_ex__`()¶
    :   helper for pickle

    `__repr__`¶

    `__setattr__`¶
    :   x.\_\_setattr\_\_(‘name’, value) <==> x.name = value

    `__sizeof__`() → int¶
    :   size of object in memory, in bytes

    `__str__`¶

High performance molecular simulation on GPUs


### Navigation

- Application Layer
- Library Layer
  - Core Objects
  - Forces
  - Integrators
  - Extras

©2015, Stanford University and the Authors.
|
Powered by Sphinx 1.3.1
& Alabaster 0.7.7
|
Page source
